# Intracystic papillary breast cancer: a clinical update

**DOI:** 10.3332/ecancer.2013.286

**Published:** 2013-01-03

**Authors:** Sara Al Reefy, Rashid Kameshki, Dhabya Al Sada, Abdullah Al Elewah, Arwa Al Awadhi, Kamil Al Awadhi

**Affiliations:** 1 King Hamad University Hospital, Muharraq, Kingdom of Bahrain; 2 Royal College of Surgeons in Ireland, Medical University of Bahrain, Kingdom of Bahrain; 3 Salmaniya Medical Complex, Manama, Kingdom of Bahrain

**Keywords:** intracystic papillary breast cancer, wide local excision, ductal carcinoma in situ, mammary ductoscopy, sentinel lymph node biopsy, radiotherapy, endocrine therapy, local recurrence

## Abstract

**Introduction:**

Intracystic (encysted) papillary cancer (IPC) is a rare entity of breast cancer accounting for approximately (1–2%) of all breast tumours [1], usually presenting in postmenopausal women and having an elusive natural history. The prediction of the biological behaviour of this rare form of breast cancer and the clinical outcome showed its overall favourable prognosis; however, its consideration as a form of ductal carcinoma *in situ* with non-invasive nature is to be reconsidered as it has been shown to present histologically with invasion of basement membrane and even metastasis [2]. The objective of this review is to shed some light on this rare, diagnostically challenging form of breast cancer, including its radiological, histological, and molecular characteristics and its pathological classification. The final goal is to optimize the clinical management including the role of sentinel lymph node biopsy (SLNB), general management with adjuvant radiotherapy (RT), mammary ductoscopy, and hormonal treatment.

**Methods:**

A literature review, facilitated by Medline, PubMed, and the Cochrane database, was carried out using the terms ‘Intracystic (encysted) papillary breast cancer’.

**Results:**

Intracystic papillary breast cancer (IPC) is best managed in the context of a multidisciplinary team. Surgical excision of the lump with margins in excess of 2 mm is considered satisfactory. Sentinel lymph node biopsy (SLNB) is recommended as data have shown the possibility of the presence of invasive cancer in the final histology. RT following IPC alone is of uncertain significance as this form of cancer is usually low grade and rarely recurs. However, if it is associated with DCIS or invasive cancer and found in young women, radiotherapy may be prudent to reduce local recurrence. Large tumours, centrally located or in cases where breast conserving surgery is unable to achieve a favourable aesthetic result, a skin sparing mastectomy with the opportunity for immediate reconstruction can be offered. Adjuvant endocrine therapy may be suggested as almost certainly these tumours are hormonal positive.

**Conclusion:**

Further research is required to determine the role of adjuvant radiotherapy and endocrine therapy in IPC. Understanding the low-grade nature of this form of breast cancer allows treatment options to be less radical and safely omitted.

## Introduction

### Diagnosis

Carter *et al* classified breast papillary lesions into invasive and non-invasive papillary carcinoma with the latter being sub-divided into two types [3]. The first one is a diffuse form, the papillary variant of DCIS, which includes medium and small ducts, and the second one is a localized form, intracystic (encysted) papillary carcinoma, which is a solitary tumour in a cystic and dilated duct. There are three main subtypes of intracystic papillary breast cancer (IPC), IPC alone (pure form), IPC with surrounding DCIS, and IPC with invasion [4].

**Figure 1:**
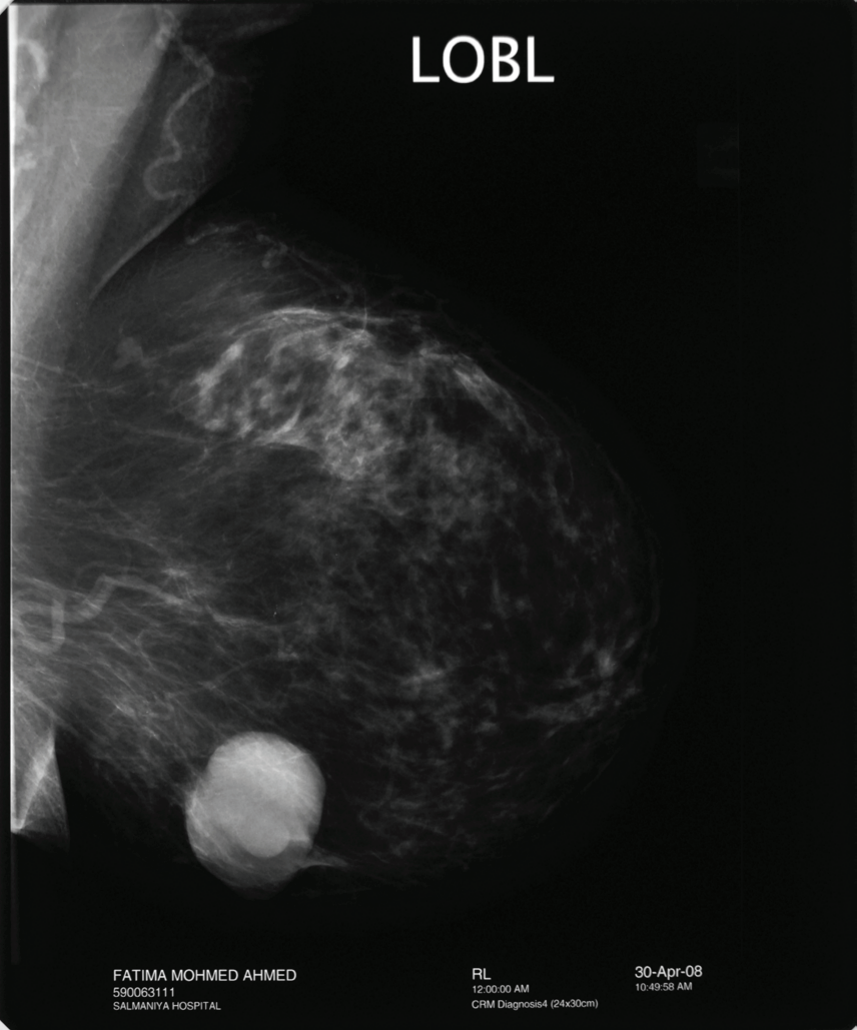
Mammogram showing well circumscribed mass: feature of intracystic papillary breast cancer

Intracystic papillary breast cancer usually occurs in elderly postmenopausal women. This form of breast cancer has a very subtle clinical presentation of a painless breast lump which could be present for a long duration or bloody nipple discharge. There are only a few cases reported in the literature of IPC occurring in younger women of less than 40 years old [4, 5]. Radiological findings may show on a mammogram as an oval or lobulated, circumscribed lesion (Figure 1) and on the ultrasound as a complex cystic mass with a solid component demonstrating vascular flow within the solid component of the cystic mass on a colour Doppler scan [6]. Such radiological characteristics should raise the suspicion of this rare form of breast cancer. Magnetic resonance imaging of the breast with contrast enhancement can further aid in the diagnosis showing enhanced cyst wall, septation, and mural nodules [7].

Cytological diagnosis may be misleading as you can aspirate the fluid within the cystic component of the lesion which could be negative for malignancy and therefore give a false negative result. Vacuum-assisted ultrasound-guided core biopsy would be superior to FNAC in suspected cases [8]. Nevertheless, the core biopsy targets the central part of the solid tumour, while the invasion usually occurs at the periphery; therefore, excision biopsy of B3 papillary lesions is recommended to exclude invasive cancer or associated adjacent DCIS [8]. Visualizing the intraductal papillary lesions by mammary ductoscopy has been described to be a valuable tool in diagnosing such lesions [9]. However, the potential of this technique in management of IPC requires further investigation [10].

## Pathological and clinical correlation

### Classification

Intracystic papillary breast cancer was originally classified as a non-invasive form of breast cancer and a variant subtype of low-grade DCIS. More recently, IPC has been repeatedly reported to occur with DCIS or invasive breast cancer in about 40% of cases [11]. Therefore, although it still carries a favourable prognosis, one must not overlook the diagnosis or under treat this highly curable condition.

Intracystic papillary breast cancer is usually of low or intermediate nuclear grade, with no evidence of necrosis, strongly estrogen receptor positive and negative for c-erbB-2. Cases of IPC associated with invasive carcinoma are found to be of nuclear grade 3 and necrosis [12].

Histologically these encysted lesions are characterized by arborisation of the fibrovascular stroma with the absence of myoepithelial layer differentiating it from benign papillary lesions [12].

The localized IPC describes an intracystic (encysted) papillary solid tumour within a cystic dilated duct, with no associated DCIS or invasion at the periphery of the tumour.

The detection of associated lesions is very important for prognostication and that the treatment decisions are tailored to associated pathology (DCIS, invasive form) [13].

For facilitating management decisions, it can be classified into three main subtypes: IPC alone, IPC with DCIS only, or IPC with invasion. Indeed, extensive sampling of the surgical pieces is considered essential. The risk for the pathologist is to miss a truly invasive component [11].

### Natural history

Although rare, this form of breast cancer has long been perceived to have good clinical prognosis. It is slow growing with a relative survival rate of 100% at 10 years and a disease-free survival rate of 91% [14]. In a recent study, which looked at the histological characteristics and myoepithelial marker of 20 IPC breast slices, features of microinvasion were noted in all cases, despite the favourable prognostic indicators The lack of myoepithelial cells is one of the important features to differentiate between invasive and in situ carcinoma of the breast. CD10 immunochemistry and comparison of its staining to those of smooth muscle actin (SMA) is used to detect myoepithelial cells [15].

Clinically, however, the largest reported study of 917 cases carried out on IPC patients from California demonstrated that the relative cumulative survival rate was no different in the group of IPC alone or associated invasive cancer (*P* = 0.18 ) followed up at 10 years [16].

## Management

Variable management strategies are considered when dealing with this rare form of breast cancer. Treatment options for the breast can involve breast conserving surgery in the form of wide local excision, with or without adjuvant RT, or mastectomy [17]. Axillary interventions, including sentinel lymph node biopsy (SLNB), and/or axillary dissection (AD) [18] and adjuvant systemic treatment which have mainly involved endocrine manipulation with Tamoxifen as this cancer seems to be almost certainly hormonal positive and HER-2 negative [13]. Despite these general principles, the optimal management of IPC remains controversial.

The low yield for metastasis and vascular invasion make chemotherapeutic intervention not mandatory. This treatment modality would be only considered in cases associated with lymphovascular invasion and can be safely omitted in IPC alone or with associated DCIS. Furthermore, the balance of benefit and risk has been influenced by the good clinical prognosis which is reported in such cases [19].

### Surgery

Irrespective of the type of surgery, the rate of recurrence and the probabilities of dying from IPC do not differ between the three groups [17].

In the cases of IPC alone, IPC with DCIS, or IPC with invasion, complete local excision of the tumour with clear margins is the recommended surgical treatment [20].

However, radical or modified radical mastectomy is not indicated for the treatment of pure IPC as this is a low-grade form of cancer, especially in elderly women in whom it so often occurs.

Nevertheless, in some cases skin-sparing mastectomy with or without immediate breast reconstruction can be offered (e.g. large tumours, inadequate margins, recurrence, and patient preference).

Axillary lymph node surgery in the form of SLNB is considered prudent in IPC; as it rarely metastasizes, in order to spare patients from the morbidity of axillary node clearance in node negative cases.

### Radiotherapy

Research to support the theory that adjuvant RT significantly reduces the risk of LR in those undergoing breast conserving surgery in cases of IPC is still ongoing.

However, many articles and published data recommend adjuvant RT for IPC associated with invasion and or DCIS [13].

Moreover, in young women (less than 50 years), with IPC alone, radiotherapy has been suggested as an adjuvant treatment [13, 17].

### Oncological therapy

Systemic adjuvant therapy has focused on endocrine manipulation, principally the use of Tamoxafin as IPC tends to be ER/PR Positive and HER 2 negative, although there is no clear indication for adjuvant endocrine therapy. Given that the overall good prognoses and that there is no adjuvant treatment which increases cancer-free survival, there is a concern for potential overtreatment in this disease [21].

Nonetheless, in young (less than 50 years old) patients with pure IPC and associated DCIS or micro-invasion, there is some evidence to suggest that adjuvant radiation and endocrine therapy should be considered to reduce the risk of local recurrence [13].
